# Influence on therapeutic outcome of platelet count at diagnosis in patients with de novo non-APL acute myeloid leukemia

**DOI:** 10.1186/s12885-023-11543-5

**Published:** 2023-10-24

**Authors:** Yujiao Zhang, Quan Wu, Baoyi Yuan, Yun Huang, Ling Jiang, Fang Liu, Ping Yan, Yongshuai Jiang, Jieyu Ye, Xuejie Jiang

**Affiliations:** 1 Department of Hematology, Nanfang Hospital, Southern Medical University, 510515 Guangzhou, Guangdong China; 2 School of Medicine, Zhengzhou University, 450001 Zhengzhou, China

**Keywords:** Acute Myeloid Leukemia, Platelet count, Therapeutic outcome, Overall survival, Relapse-free survival

## Abstract

**Background:**

Platelet (PLT) count at diagnosis plays an important role in cancer development and progression in solid tumors. However, it remains controversial whether PLT count at diagnosis influences therapeutic outcome in patients with non-acute promyelocytic leukemia (APL) acute myeloid leukemia (AML).

**Methods:**

This study analyzed the relationship between PLT count at diagnosis and genetic mutations in a cohort of 330 newly diagnosed non-APL AML patients. The impact of PLT count on complete remission, minimal residual disease status and relapse-free survival (RFS) were evaluated after chemotherapy or allogeneic hematopoietic stem cell transplantation (allo-HSCT).

**Results:**

Our studies showed that patients with DNMT3A mutations have a higher PLT count at diagnosis, while patients with CEBPA biallelic mutations or t(8;21)(q22; q22) translocation had lower PLT count at diagnosis. Furthermore, non-APL AML patients with high platelet count (> 65 × 10^9^/L) at diagnosis had worse response to induction chemotherapy and RFS than those with low PLT count. In addition, allo-HSCT could not absolutely attenuated the negative impact of high PLT count on the survival of non-APL AML patients.

**Conclusion:**

PLT count at diagnosis has a predictive value for therapeutic outcome for non-APL AML patients.

**Supplementary Information:**

The online version contains supplementary material available at 10.1186/s12885-023-11543-5.

## Introduction

Acute myeloid leukemia (AML) is one of the most common adult hematopoietic malignancies with poor prognosis [1, 2]. 70% of AML patients achieve complete morphologic remission after standard “3 + 7” induction treatment, most patients with complete remission (CR) relapse and progress to refractory leukemia after consolidation therapy [3, 4]. The persistence of minimal residual disease (MRD) is a risk factor for leukemic recurrence in AML patients after chemotherapy [5, 6]. Allogeneic hematopoietic stem cell transplantation (allo-HSCT) was necessary to decrease the probability of relapse for AML patients [7, 8]. Therefore, it was important to identify the adverse outcome-related risk factors at diagnosis in AML.

Cytogenetics, molecular abnormalities and epigenetic alterations have been acknowledged as the most important prognostic factors in AML patients [1, 2, 4, 9]. In addition, clinical characteristics contribute to chemotherapeutic response and survival in these patients; for instance, high white blood cell count (WBC) is associated with more probability of early mortality and occurrence of central nervous system leukemia [10, 11]. Clinically, the majority of AML patients have thrombocytopenia, leukocytosis and anemia at diagnosis, and only a small number of patients have normal or high platelet (PLT) count [12, 13]. Studies showed that PLTs increased the resistance of colon and ovarian cancer cell lines to 5-fluorouracil and paclitaxel [14, 15]. Increasing evidence suggested that PLTs played a predominant role in colon and breast cancer cells metastasis to lung and brain [16, 17]. Thrombocytosis was considered as an adverse-risk factor in lung, gastric, colon, breast and kidney cancers [18–21]. Reportedly, platelet microparticles (PMPs) in AML was important in leukemic development and contributed to chemotherapeutic resistance [22, 23]. A clinical trial showed that AML patients with a medium PLT count of 50–120 × 10^9^/L had longer overall survival (OS) and disease-free survival (DFS) than the other patients [24]. Other studies showed that low PLT count were associated with better survival in intermediate-risk AML patients [24, 25]. However, the influence of high PLT count on therapeutic outcome remains obscure in AML patients.

In this study, we investigated the relationship between clinic characteristics and PLT count, analyzed the impact of PLT count on therapeutic outcome and MRD status, followed up their survival after chemotherapy or allo-HSCT in newly diagnosed non-APL AML patients.

## Patients and methods

### Patients

**Fig. 1:**
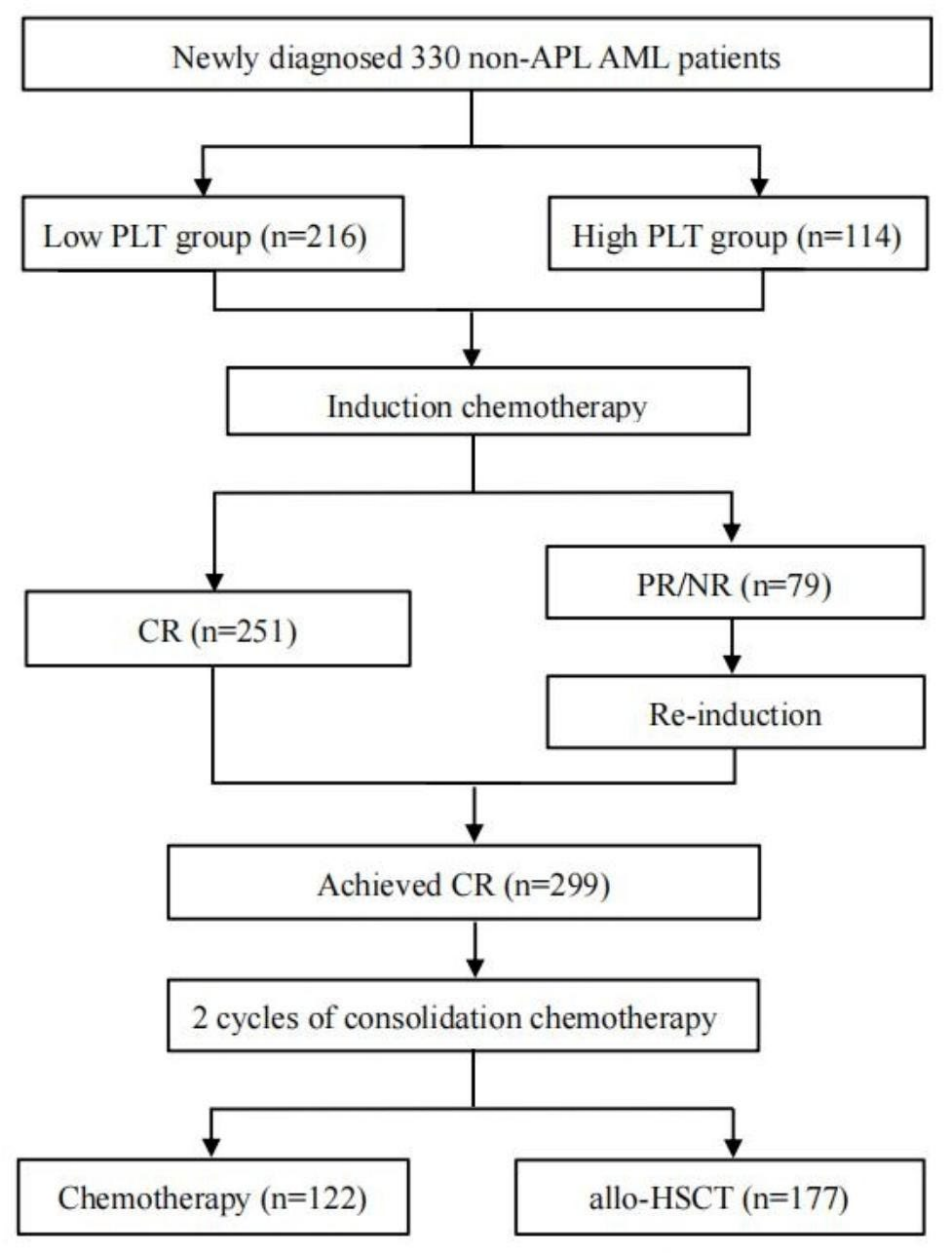
Patients flow diagram

This retrospective study enrolled 330 newly diagnosed de novo non-APL AML patients aged 16–65 years in Nanfang Hospital (Guangzhou, China) from June 2018 to December 2020. The inclusion criteria were as following: (1) age 16–65 years, (2) de novo AML, (3) standard “3 + 7” induction regime and (4) at least four courses of chemotherapy with regular follow-up. The exclusion criteria were as following: (1) preceding hematological disorders, (2) therapy-related AML or (3) other carcinomas. Patients were diagnosed according to the French-American-British (FAB) and 2016 revision of the World Health Organization classification of myeloid neoplasms [26]. Molecular mutational abnormalities were detected by next-generation sequencing (NGS). The designed panel included: CEBPA, FLT3, KIT, NPM1, ASXL1, RUNX1, BCOR, EZH2, SF3B1, SRSF2, U2AF1, ZRSR2, DNMT3A, GATA2, IDH1, IDH2, NRAS, MLL, KRAS, PHF6, TET2, TP53, WT1, STAG2, SETBP1, ETV6, JAK2, CALR, MPL, SH2B3, and CSF3R (Supplement. Table S1). Risk groups were classified according to the 2022 European Leukemia Net (ELN) guideline [2]. The study protocol was reviewed and approved by ethics committee, and patient flow diagram was shown in Fig. 1.

### Treatments

First, patients were treated with standard induction chemotherapy based on the “3 + 7” regimen, including idarubicin (IDA 10 mg/m^2^, days 1–3) or daunorubicin (DNR 60 mg/m^2^, days 1–3) and cytarabine (Ara-C 100 mg/m^2^, days 1–7). Bone marrow (BM) aspiration was performed after 14–21 days post-induction chemotherapy to evaluate the treatment response. Patients with CR continued to receive two cycles of consolidation chemotherapy based on high-dose cytarabine (HD-Ara-C 2 g/m^2^, twice, days 1–3). Patients without CR received the second cycle of induction chemotherapy based on high-dose Ara-C (Ara-C 2 g/m^2^ plus cladribine 5 mg/m^2^, days 1–5 and G-CSF 300 ug days 0–5). Routine blood tests were performed to provide necessary supportive treatment. Tyrosine kinase inhibitors or FLT3 inhibitors were added to the induction and consolidation treatments in Philadelphia or FLT3-internal tandem duplication (ITD)-positive AML patients. After CR, all patients received two cycles of cytarabine-based consolidation chemotherapy. For adverse-risk patients, allo-HSCT was administrated after two cycles of consolidation chemotherapy, except for those without HLA-matched donors or refusing haploidentical transplantation. For favorable and intermediate-risk patients, MRD status after two cycles of consolidation chemotherapy was a critical indicator to determine subsequent treatment strategies. Patients with negative MRD (MRD-) continue with two cycles of consolidation chemotherapy and those with positive MRD (MRD+) underwent allo-HSCT.

### Allo-HSCT

As described previously [27], there were two alternative myeloablative conditioning regimens in our center, including busulfan (Bu 3.2 mg/kg/day, -7 to -4 days) + cyclophosphamide (Cy 60 mg/kg/day, -3 to -2 days) and Bu (3.2 mg/kg/day, -6 to -3 days) + fludarabine (Flu 30 mg/m^2^, -6 to -2 days). Graft-versus-host disease (GVHD) prophylaxis was regularly administered, such as cyclosporine A (CsA) plus methotrexate (MTX) in HLA-matched sibling donor transplant, CsA + MTX + antithymocyte globulin and/or mycophenolate in HLA-matched unrelated donor and haploidentical transplants. CsA was gradually withdrawn after 30 days post-transplantation, and donor lymphocyte infusion was applied after 60 days post-transplantation in patients without GVHD. Effective regiments were used to keep GVHD under control, such as methylprednisolone, CsA, CD25 monoclonal antibody or combined with other immunosuppressive agents for acute GVHD, corticosteroids and CsA for chronic GVHD.

### Definition of clinical end points

Treatment response was assessed by routine blood tests and BM morphology according to standardization response criteria. CR was defined as < 5% BM leukemic blasts with normal maturation of all cell lineages [28]. In addition, recovery of neutrophils (≥ 1500/µl) and PLTs (≥ 100,000/µl) in peripheral blood was mandatory, with no evidence of circulating blasts and/or extramedullary leukemia. Relapse was defined as the re-occurrence of 5% leukemic blasts in BM, re-appearance of circulating blasts or development of extramedullary leukemia [28]. MRD was performed by multiparametric flow cytometry to detect abnormal leukemia populations with leukemia-associated immunophenotypes in total CD45 + cells in patients with CR before each cycle of consolidation therapy. MRD- was defined as the detection of < 0.1% abnormal cells, and MRD + was defined as the detection of ≥ 0.1% abnormal cells. Patients achieved CR were followed up for 2 years to calculate their relapse-free survival (RFS). RFS was measured from the date of first CR (CR1) until death, the first relapse, or the last follow-up in continuous CR.

### Statistical analysis

All clinical data were analyzed using SPSS (SPSS, Chicago, IL), Prism9 (GraphPad Software, La Jolla, CA). Median values and ranges were used for continuous variables and percentages for categorical variables. Groups were compared using the Pearson chi-square test or Fisher’s exact test for categorical variables and Mann-Whitney U tests for continuous variables. The discriminatory power of PLT count value to predict CR was assessed by estimating the area under the receiver operating characteristic (ROC) curve (AUC). The optimal cut-off value was determined by maximizing sensitivity and specificity and their 95% confidence intervals (CIs). Cox proportional hazards regression models were used to determine the influence of PLT count on RFS in AML patients after chemotherapy or allo-HSCT, and the results are expressed as hazard ratio (HR) with 95% CI. All statistical tests were 2-sided, and a *P* value of < 0.05 was considered statistically significant.

## Results

### Clinical characteristics of AML patients

**Fig. 2:**
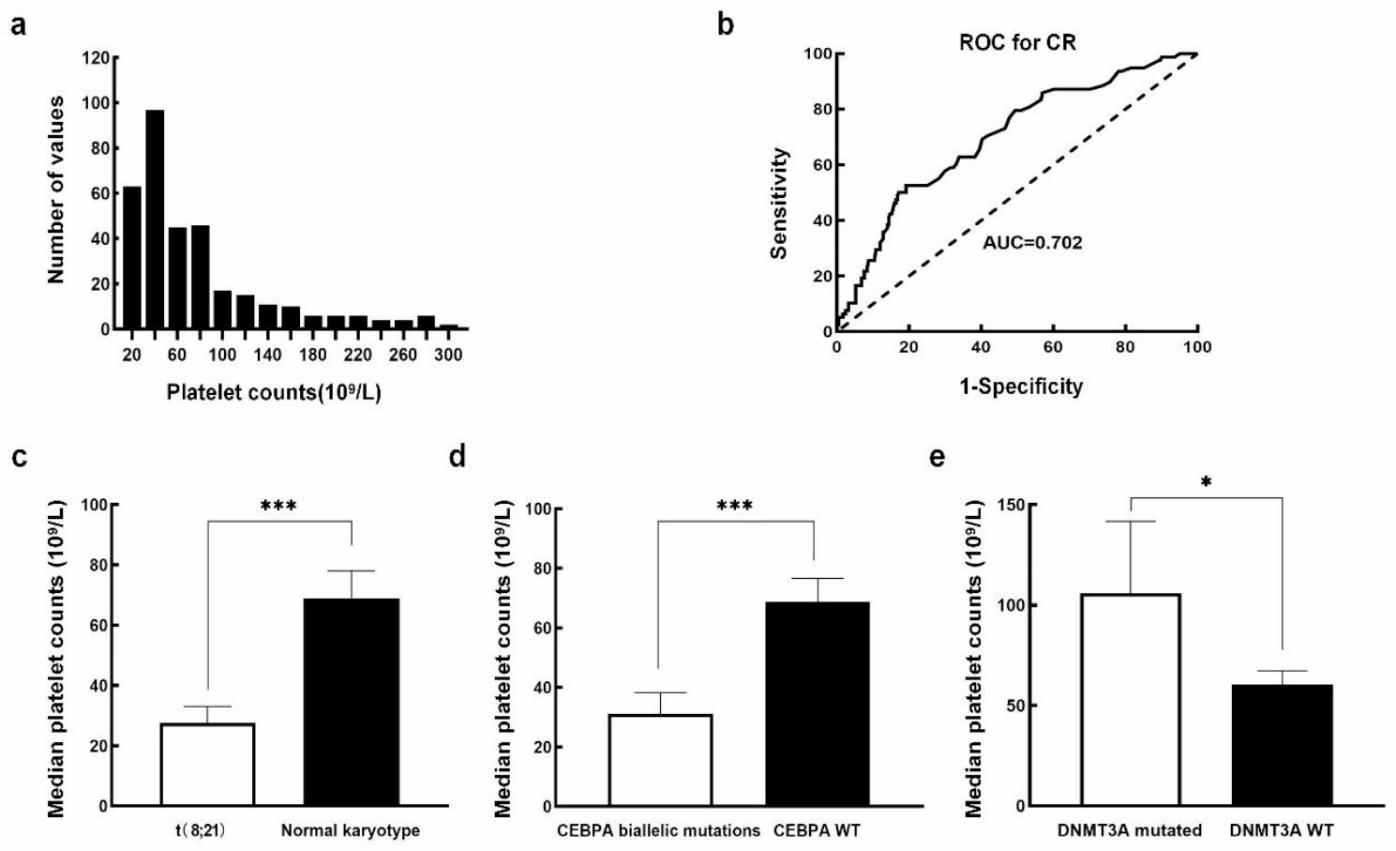
**(a)** Distribution of PLT counts in 330 newly diagnosed AML patients. **(b)** ROC curve analysis for initial PLT count > 65 × 10^9^/L. **(c)** Comparison of PLT counts between patients with t(8;21) (n = 34) and those with normal karyotypes (n = 217). **(d)** Comparison of PLT counts between patients with mutated CEBPA biallelic mutation (n = 29) and wild-type mutation (n = 301). (e) Comparison of PLT counts between patients with mutated DNMT3A (n = 33) and wild-type DNMT3A (n = 297). *P < 0.05, **P < 0.01,***P < 0.001 PLT: platelet; AML: acute myeloid leukemia; ROC: Receiver operating characteristic

A cohort of 330 patients were included in the study. PLT counts ranged from 6 to 314, with a median of 42 × 10^9^/L. The distribution of PLT counts did not suggest apparent grouping (Fig. 2a). Based on CR after the first cycle of induction chemotherapy, the cut-off value of PLT count for therapeutic outcome in AML patients according to the ROC curve analysis was 65 × 10^9^/L (Fig. 2b). Therefore, the patients were divided into low PLT count group (≤ 65 × 10^9^/L, n = 216, 65.5%) and high PLT count group (> 65 × 10^9^/L, n = 114, 34.5%). The clinical characteristics of these patients were shown in Table 1. Patients with high PLT count had higher hemoglobin levels and more megakaryocytes (MKs) in the BM (*P* = 0.006 and 0.001). Cytogenetics and molecular abnormalities were compared between both groups. As shown in Table 2, the high PLT count group had more patients with DNMT3A mutation, and the low PLT count group had more patients with t(8;21)(q22;q22) translocation and CEBPA biallelic mutation. In addition, we found that PLT counts in patients with t(8;21)(q22;q22) were lower than in those with normal karyotype (27 × 10^9^/L vs. 68 × 10^9^/L, *P* = 0.000, Fig. 2c). Patients with CEBPA biallelic mutations had lower PLT count (31 × 10^9^/L, *P* = 0.013, Fig. 2d), and those with DNMT3A mutations had higher PLT count (105 × 10^9^/L, *P* = 0.000, Fig. 2e), as compared with CEBPA and DNMT3A wild-type at diagnosis (68 × 10^9^/L and 60 × 10^9^/L).

**Table 1:** Patient characteristics

Characteristic	Low PLT (≤ 65 × 10^9^/L) group (n = 216)	High PLT (> 65 × 10^9^/L) group (n = 114)	*P* value
Median age (range)	39 (17–65)	44 (18–65)	0.078
Gender, n (%)			0.190
Male	103 (47.7)	63 (55.3)	
Female	113 (52.3)	51 (44.7)	
Median WBC count (range), ×10^9^/L	18.5 (0.75–425.4)	19.2 (1.25–228.0)	0.908
Median hemoglobin (range), g/L	75 (26–142)	86 (39–208)	0.006
Megakaryocytes, n/1.5 × 3 cm^2^	2 (0-388)	16.5 (0-920)	0.001
Median blasts in BM (range), %	50.5 (25–91)	63.5(21–95)	0.104
FAB subtypes, n (%)			
M0	0	0	
M1	10 (4.6)	1 (0.9)	0.138
M2	136 (63.0)	61 (53.5)	0.096
M4	23 (10.6)	9 (7.9)	0.422
M5	41 (18.9)	36 (31.6)	0.010
M6	1 (0.5)	0	1.000
M7	0	0	
Unclassified	5 (2.4)	7 (6.1)	0.078
ELN risk group, n (%)			0.001
Favorable	99 (45.8)	23 (20.2)	
Intermediate	54 (25.0)	42 (36.8)	
Adverse	63 (29.2)	49 (43.0)	
Induction chemotherapy, n (%)			0.122
IA	202 (93.5)	99 (86.8)	
DA	11 (5.1)	8 (7.1)	
HA	3 (1.4)	7 (6.1)	
Extramedullary involvement, n (%)	3 (1.4)	2 (1.8)	0.796

**Table 2:** Cytogenetics and molecular abnormalities between low and high PLT groups

Cytogenetics and molecular abnormalities	Low PLT (≤ 65 × 10^9^/L) group (n = 216)	High PLT (> 65 × 10^9^/L) group (n = 114)	*P* value
Cytogenetics, n (%)			
t(8;21)(q22; q22)	33 (15.3)	1 (0.9)	0.001
inv(16)(p13.1; q22)	11 (5.1)	2 (1.8)	0.138
t(9;11)(p21.3; q23.3)	0	1 (0.9)	0.745
t(6;9)(p23.3; q34.1)	1 (0.5)	1 (0.9)	1.000
t(v;11q23.3)	6 (2.8)	3 (2.6)	0.938
t(9;22)(q34.1; q11.2)	1 (0.5)	0	1.000
t(8;16)(p11.2; p13.3)	0	0	
inv(3)(q21.3q26.2)	0	1(0.9)	0.745
–5, 5q–, − 7, − 17	7 (3.2)	4 (3.5)	0.897
Complex karyotype	4 (1.8)	3 (2.6)	0.948
Normal cytogenetics	133 (61.6)	82 (70.7)	0.060
Other nondeified cytogenetics	22 (10.2)	16 (14.0)	0.297
Genetic mutations, n (%)			
NPM1	29 (13.4)	21 (18.4)	0.229
CEBPA^bi^	25 (11.6)	4 (3.5)	0.013
CEBPA^smbZIP^	10 (4.6)	6 (5.3)	0.920
FLT3-ITD	26 (12.0)	22 (19.3)	0.075
TP53	1 (0.5)	1(0.8)	1.000
ASXL1	24 (11.1)	18 (15.8)	0.225
RUNX1	16 (7.4)	10 (8.7)	0.662
EZH2	14 (6.5)	8 (7.0)	0.853
BCOR	6 (2.8)	6 (5.3)	0.251
SF3B1	2 (1.0)	1 (0.9)	1.000
SRSF2	3 (1.4)	0	0.513
STAG2	3 (1.4)	2 (1.8)	1.000
U2AF1	4 (1.9)	1 (0.9)	0.829
ZRSR2	1 (0.5)	0	1.000
DNMT3A	13 (6.0)	20 (17.5)	0.001
No mutations detected	24 (11.1)	18 (15.7)	0.225

CEBPA^bi^, biallelic mutations of the CEBPA gene; CEBPA^smbZIP^, monoallelic mutations of the CEBPA gene in C-terminal DNA-binding or basic leucine zipper region

### Impact of platelet count on induction chemotherapy response in AML

After the first course of induction chemotherapy, 85.6% (185/216) of the patients in low PLT group achieved CR while 57.9% (66/114) in high PLT group (*P* = 0.000). Among risk groups based on 2022 ELN classification, there were significant difference in CR rate between low and high PLT group in intermediate-risk group (75.9% vs. 47.6%, *P* = 0.004) and adverse-risk group (81.0% vs. 49.0%, *P* < 0.001) except favorable-risk group (93.9% vs. 95.7%, *P* = 0.750).

**Fig. 3:**
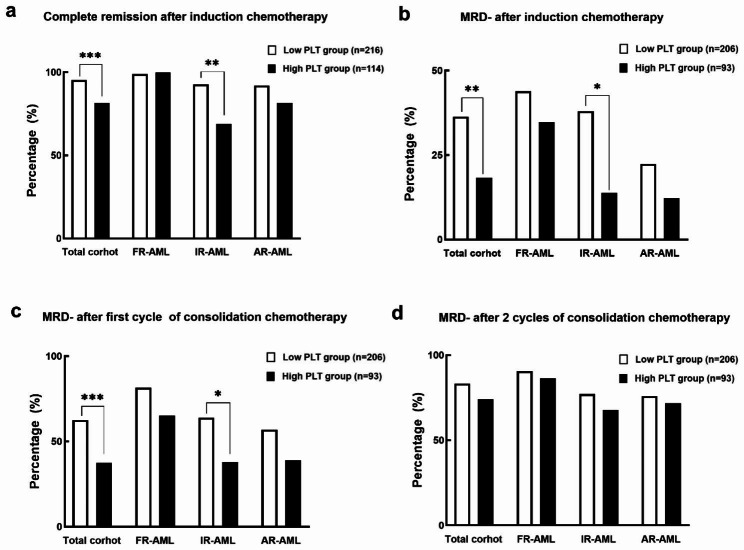
**(a)** The proportion of CR after 1–2 cycles of induction chemotherapy between low and high PLT groups in the whole cohort of AML, favorable-risk AML (FR)-AML, intermediate-risk AML (IR-AML) and adverse-risk AML (AR-AML) patients. **(b)** The proportion of MRD-negative CR (CR/MRD-) after induction chemotherapy between low and high PLT groups in the whole cohort of AML, FR-AML, IR-AML and AR-AML patients. **(c)** The proportion of CR/MRD- after the first cycle of consolidation of chemotherapy between low and high PLT groups in the whole AML and different subgroups. **(d)** The proportion of CR/MRD- after two cycles of consolidation of chemotherapy between low and high PLT groups in the whole cohort and different subgroups. *P < 0.05, **P < 0.01,***P < 0.001 CR: complete remission; PLT: platelet; AML: acute myeloid leukemia; FR: favorable-risk; IR: intermediate-risk; AR: adverse-risk; MRD: minimal residual disease

Patients without CR received the second cycle of induction chemotherapy (n = 79); 95.4% (206/216) of patients in the low PLT group achieved CR as compared to 81.6% (93/114) in the high PLT group (*P* = 0.000, Fig. 3a). After two cycles of induction chemotherapy, patients in the low PLT group had a higher CR rate than the high PLT group in intermediate-risk group (92.6% vs. 69.0%, *P* = 0.003, Fig. 3a). However, the CR rates were not significantly different between the low and high PLT groups in favorable-risk group (99.0% vs. 100%, *P* = 0.628, Fig. 3a) and adverse-risk group (92.1% vs. 81.6%, *P* = 0.098, Fig. 3a).

### Impact of platelet count on MRD status in AML

After induction chemotherapy, more patients in the low PLT group (75/206) achieved CR/MRD- compared with patients in the high PLT group (17/93) (36.4% vs. 18.3%, *P* = 0.002, Fig. 3b). In the intermediate-risk, there were more patients with CR/MRD- in the low PLT group than in the high PLT group (38.0% vs. 13.8%, *P* = 0.022, Fig. 3b). There was no difference in the proportion of CR/MRD- between patients with low and high PLT count in favorable-risk group (43.9% vs. 34.8%, *P* = 0.427, Fig. 3b) and adverse-risk groups (22.4% vs. 12.2%, *P* = 0.194, Fig. 3b). After the first cycle of consolidation chemotherapy, the low PLT group had more patients with CR/MRD- than the high PLT group in the whole cohort (62.5% vs. 37.5%, *P* = 0.000, Fig. 3c). This finding was also observed in the intermediate-risk group (64.0% vs. 37.9%, *P* = 0.025, Fig. 3c) but not in favorable-risk (81.6% vs. 65.2%, *P* = 0.085, Fig. 3c) and adverse-risk groups (56.9% vs. 39.0%, *P* = 0.081, Fig. 3c). After two cycles of consolidation chemotherapy, there were no significant differences in the proportion of CR/MRD- between the low and high PLT groups in the whole cohort (83.3% vs. 74.2%, *P* = 0.070, Fig. 3d), including favorable-risk (90.6% vs. 86.4%, *P* = 0.551, Fig. 3d), intermediate-risk (77.1% vs. 67.9%, *P* = 0.378, Fig. 3d) and adverse-risk groups (75.9% vs. 71.8%, *P* = 0.653, Fig. 3d).

### Impact of platelet count on relapse-free survival in AML patients treated with chemotherapy

**Fig. 4:**
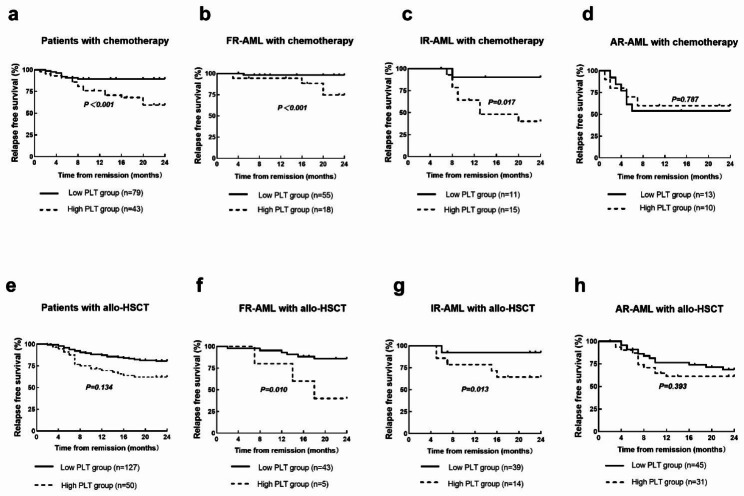
**(a-d)** For whole AML patients treated with chemotherapy only, the 2-year RFS was compared between low- and high-PLT groups; (a) The 2-year RFS between low- and high-PLT groups in AML; **(b)** In favorable-risk AML, the 2-year RFS between low- and high-PLT groups; **(c)** In intermediate-risk AML, the 2-year RFS between low- and high-PLT groups; **(d)** In adverse-risk AML, the 2-year RFS between low- and high-PLT groups in AML; **(e-h)** For whole AML patients treated with allo-HSCT, the 2-year RFS was compared between low- and high-PLT groups; **(e)** The 2-year RFS between low- and high-PLT groups in AML; **(f)** In favorable-risk AML, the 2-year RFS between low- and high-PLT groups; **(g)** In intermediate-risk AML, the 2-year RFS between low- and high-PLT groups; **(h)** In adverse-risk AML, the 2-year RFS between low- and high-PLT groups in AML

After 1–2 cycles of induction chemotherapy, 299 patients achieved CR. Among them, 40.8% (122/299) of patients received chemotherapy alone; here, patients with low PLT count had better 2-year RFS than those with high PLT count (89.9% vs. 58.1%, *P* = 0.000, Fig. 4a). In the subgroup analysis, a better RFS was also observed in favorable-risk group (98.2% vs. 66.7%, *P* = 0.000, Fig. 4b) and intermediate-risk group (90.0% vs. 46.7%, *P* = 0.017, Fig. 4c), but not in adverse-risk group (53.8% vs. 60.0%, *P* = 0.787, Fig. 4d).

### Impact of platelet count on relapse-free survival in AML patients treated with allo-HSCT

There were 177 (59.2%, 177/299) patients with CR received allo-HSCT. Clinic and transplant characteristics of these patients were shown in Table 3. The patients with low PLT count had better 2-year RFS than those with high PLT count (82.7% vs. 60.0%, *P* = 0.001, Fig. 4e). In the subgroup analyses, patients with low PLT count had better 2-year RFS than those with high PLT count in favorable-risk (86.0% vs. 40.0%, *P* = 0.010, Fig. 4f) and intermediate-risk group (92.3% vs. 64.3%, *P* = 0.013, Fig. 4g). However, there weren’t different 2-year RFS between patients with low and high PLT count in adverse-risk group (71.7% vs. 61.3%, *P* = 0.393, Fig. 4h).

**Table 3:** Clinic and transplant characteristics in Allogeneic hematopoietic stem cell transplantation patients

Characteristic	Low PLT (≤ 65 × 10^9^/L) group (n = 127)		High PLT (> 65 × 10^9^/L) group (n = 50)	*P* value
Median patients age (range)		39 (17–65)	38 (18–61)	0.725
Gender, n (%)				0.318
Male		58 (45.7)	27 (54.0)	
Female		69 (54.3)	23 (46.0)	
Type of donor, n (%)				0.892
MSD		53 (41.7)	19 (38.0)	
HID		72 (56.7)	30 (60.0)	
MUD		2 (1.6)	1 (2.0)	
Stem cell source, n (%)				0.219
PBSC		48 (37.8)	14 (28.0)	
PBSC + BM		79 (62.2)	36 (72.0)	
Conditioning regimen, n (%)				0.212
Bu-Cy		91 (71.6)	31 (62.0)	
Bu-Flu		36 (28.4)	19 (38.0)	
GVHD prophylaxis, n (%)				0.863
CsA + MTX		53 (41.7)	19 (38.0)	
CsA + MTX + ATG		6 (4.7)	2 (4.0)	
CsA + MTX + ATG + MMF		68 (53.6)	29 (58.0)	

MSD HLA-matched sibling donor, MUD HLA-matched unrelated donor, HID haploidentical related donors, PBSC peripheral blood stem cell, BM bone morrow, Bu busulfan, Cy cyclophosphamide, Flu fludarabine, GVHD graft-versus-host disease, CsA cyclosporine A, MTX methotrexate, ATG antithymocyte globulin, MMF mycophenolate

### Multivariate analysis of relapse-free survival

The multivariate analyses of RFS were presented in Table 4. Cox proportional hazards regression analysis revealed that low PLT count (≤ 65 × 10^9^/L, HR 0.334, 95% CI 0.197–0.565, *P* = 0.001), WBC < 20 × 10^9^/L (HR 0.321, 95% CI 0.187–0.550, *P* = 0.001) and ELN favorable risk (HR 0.408, 95% CI 0.207–0.804, *P* = 0.010) were found to be significantly associated with an increasing RFS in the whole AML group. For patients in favorable-risk group, low PLT count (≤ 65 × 10^9^/L) was an independent protective factor for RFS (HR 0.068, 95% CI 0.020–0.237, *P* = 0.001). In addition, low platelet count (≤ 65 × 10^9^/L, HR 0.084, 95% CI 0.021–0.342, *P* = 0.001) had a beneficial association with RFS in intermediate-risk group.

**Table 4:** Multivariate Analysis for Relapse-free Survival

Variable	HR (95% CI)	*P* value	
Whole cohort			
Age (< 50y vs. > 50y)	0.996 (0.974–1.018)	0.707	
Gender (Male vs. Female)	1.254 (0.759–2.070)	0.377	
WBC (×10^9^/L, < 20 vs. > 20)	0.321 (0.187–0.550)	0.001	
HGB (g/L, < 90 vs. > 90)	1.462 (0.863–2.476)	0.158	
PLT (×10^9^/L, < 65 vs. > 65)	0.334 (0.197–0.565)	0.001	
Cycles to achieve CR (2 vs. 1)	0.778 (0.425–1.423)	0.415	
ELN favorable (yes vs. no)	0.408 (0.207–0.804)	0.010	
Chemotherapy vs. Allo-HSCT	1.268 (0.717–2.241)	0.415	
Favorable-risk group			
Age (< 50y vs. > 50y)	1.012 (0.970–1.056)	0.575	
Gender (Male vs. Female)	0.858 (0.294–2.509)	0.780	
WBC (×10^9^/L, < 20 vs. > 20)	0.386 (0.138–1.080)	0.070	
HGB (g/L, < 90 vs. > 90)	0.809 (0.227–2.885)	0.774	
PLT (×10^9^/L, < 65 vs. > 65)	0.068 (0.020–0.237)	0.001	
CEBPA biallelic (yes vs. no)	0.311 (0.049–1.953)	0.231	
t(8;21)(q22; q22) (yes vs. no)	0.495 (0.053–0.917)	0.321	
Intermediate-risk group			
Age (< 50y vs. > 50y) Gender (Male vs. Female) WBC (×10 ^9^/L, < 20 vs. > 20) HGB (g/L, < 90 vs. > 90) PLT (×10^9^/L, < 65 vs. > 65) FLT3-ITD + vs. FLT3-ITD-	0.969 (0.921–1.019) 1.049 (0.329–3.345) 0.307 (0.229–0.599) 2.301 (0.644–8.223) 0.084 (0.021–0.342) 1.830 (0.470–7.126)	0.225 0.936 0.010 0.200 0.001 0.384	
Normal cytogenetics vs. other cytogenetics	0.355 (0.113–1.119)	0.077	
Chemotherapy vs. Allo-HSCT	1.547 (0.515–4.645)	0.437	

## Discussion

AML is a hematological malignancy with significant clinical heterogeneity. Cytogenetic abnormalities and molecular mutations are critical indicators for the prognostic stratification of AML patients, which can help formulate an optimal therapy strategy [29–31]. In addition, clinical characteristics at diagnosis also contribute to chemotherapeutic response and survival in AML patients [10]. In this study, our data showed that higher PLT count was associated with worse response to induction chemotherapy, fewer proportion of CR/MRD- and shorter RFS in AML patients. We further noted that these effects were more profound in intermediate-risk patients than favorable and adverse-risk AML patients. These findings were consistent with other investigations that lower PLT count predicted better survival in intermediate-risk group [24, 32]. Moreover, hyperleukocytosis defined as WBC > 100 × 10^9^/L at diagnosis was demonstrated to relate with increasing early mortality in AML patients [10, 33], which prognostic significance in RFS is not widely recognized [34, 35]. Our data showed that WBC < 20 × 10^9^/L was a beneficial factor for RFS in AML patients. It was also reported that WBC ≥ 20 × 10^9^/L was correlated with decreasing EFS in newly diagnosed cytogenetically normal AML patients [36].

Some research showed that AML patients with medium PLT count at diagnosis in the range of 50–120 × 10^9^/L had longer OS and DFS than the other patients [24]. Others reported that pretreatment PLT count > 130 × 10^9^/L was an unfavorable prognostic factor for chemotherapy response and prognosis in AML patients [32]. Although cut-off value of PLT count was various in different studies, these clinical data demonstrated that higher PLT count harbored a negative impact on survival of AML patients. However, the relationship between PLT count and therapeutic outcome in different risk groups was unclear. About 50% AML patients are classified as intermediate-risk group based on ELN risk classification, which 4-year OS was no more than 50% [37]. Therefore, it is critical to identify novel risk factors for patients in intermediate-risk group who fail to induction therapy. Our study demonstrated that PLT count was considered as a valuable indicator to predict therapeutic response and RFS of AML patients in intermediate-risk group.

Platelets have been reported to play a pivotal role in cell proliferation, metastasis, drug resistance in lung and ovarian cancers [38, 39]. Higher PLT count confers poor prognosis in many cancer types, including colon, lung, ovary, and stomach [19, 20, 40, 41]. A variety of substances stored and secreted by platelets had effect on proliferation of leukemic cells, such as platelet-derived growth factor, vascular endothelial growth factor, transforming growth factor-β and serotonin [42–46]. It was shown that PMPs could be internalized by AML cells, which transferred microRNAs in relation to chemotherapy resistance from platelets to leukemic cells via PMPs internalization [22, 47–49]. Our study showed that PLT count were negatively associated with chemosensitivity of AML patients in intermediate-risk group, but the mechanisms need to be further explored.

Studies showed that cytogenetic or molecular abnormalities had an influence on proliferation and differentiation of MKs as well as platelets production in AML patients [32, 50]. The thrombopoietin (TPO)/myeloproliferative leukemia virus oncogene (MPL) pathway plays a critical role in both normal and malignant hematopoiesis and megakaryopoiesis [51, 52]. Furthermore, TPO/MPL pathway are involved in the interaction of human leukemic stem cells (LSCs) with hematopoietic microenvironment [53, 54]. Upregulation of the TPO/MPL signaling pathway protects the human LSCs from chemotherapy, which results in chemoresistance and recurrence [54]. It was reported that TPO/MPL signaling was up-regulated in DNMT3A mutated AML patients with high PLT count and poor prognosis [55]. Our data showed that high PLT count at diagnosis was found in DNMT3A mutated AML, which was more probability detected in M5 subtype [56, 57]. Interestingly, it was reported that high expression of MPL on blasts in AML with t(8;21) led to severe thrombocytopenia by scavenging TPO [25, 58], which was consistent with our results.

There were limited numbers of patients in our single-center retrospective study, it wasn’t found that normal or elevated platelet counts were frequently detected in AML patents with some genetic mutations or cytogenetic abnormalities as RUNX1 and chromosome 3q abnormalities, which were reported in other studies [59, 60]. Otherwise, the mechanism of influence on therapeutic outcome by platelets wasn’t explored, investigations was needed in our further study.

In conclusion, our study demonstrated that higher platelet count at diagnosis was related to worse therapeutic outcome and shorter RFS in AML patients, especially in intermediate-risk AML patients. Further mechanistic investigations are needed to provide novel potential targets for AML patients.

### Electronic supplementary material

Below is the link to the electronic supplementary material.

Supplementary Material 1

## Data Availability

The datasets used and/or analysed during the current study available from the corresponding author on reasonable request.
