# Supplementary material for: Influence on therapeutic outcome of platelet count at diagnosis in patients with de novo non-APL acute myeloid leukemia
Source: BMC Cancer. 2023 Oct 24;23:1030. doi: 10.1186/s12885-023-11543-5 (PMC10598966; doi:10.1186/s12885-023-11543-5)
Supplement: Supplementary file 1 — Supplementary Material 1 [file 12885_2023_11543_MOESM1_ESM.docx]

**Table S1.**

**List of molecular mutations by next-generation sequencing in newly diagnosed AML patients**

| ABCA12 | CCND3 | DIS3 | GATA3 | MAP2K1 | NUP98 | SETD2 |
| --- | --- | --- | --- | --- | --- | --- |
| ABL1 | CD101 | DNM2 | GNAS | MDM2 | PAX5 | SF1 |
| ABL2 | CD123 | DNMT1 | HMGA2 | MEF2B | PDGFRA | SF3A1 |
| ACTR5 | CD79b | DNMT3A | HMGB1 | MLL | PDGFRB | SF3B1 |
| AKT1 | CDC27 | EBF1 | HRAS | MLL2 | PHF6 | SH2B3 |
| ALK | CDK4 | ECT2L | ID2 | MLL3 | PI3KCA | SOCS1 |
| ANK3 | CDKN2A | EED | IDH1 | MLL5 | PICALM | SOX4 |
| ARID1A | CDKN2B | EGFR | IDH2 | MMD2 | PKM2 | SRSF2 |
| ARID2 | CEBPA | EP300 | IKZF | MN1 | PRDM1 | SSPO |
| ASXL1 | CHD1 | EPHA7 | IL7R | MPL | PRMT5 | STAG1 |
| ATM | CRBN | ERG1 | IRF4 | MTAP | PRPF40B | STAG2 |
| ATRX | CREBBP | ETV6 | IRF6 | MUM1 | PTEN | STAT3 |
| BAFF | CRLF2 | EVI1 | JAK1 | MYC | PTPN11 | STAT5a |
| BCL2 | CSF1R | EZH2 | JAK2 | MYD88 | PU.1 | STAT5b |
| BCL6 | CSF3R | FAM46C | JAK3 | MYH11 | RAD21 | SUZ12 |
| BCOR | CUX1 | FAT1 | KDM2B | NF1 | RB1 | SYK |
| BIRC3 | CXCL12 | FAT4 | KDM5A | NFKB2 | RELN | TCF3 |
| BRAF | CXCR4 | FBXW7 | KDM6A | NOTCH1 | RHOA | TERC |
| CALR | CYLD | FGFR3 | KIT | NOTCH2 | ROS1 | TET1 |
| CARD11 | DDB1 | FLT3 | KRAS | NPM1 | RUNX1 | TET2 |
| CBL | DDX18 | GATA1 | LMO1 | NRAS | RUNX2 | TIM-3 |
| CCND1 | DDX3X | GATA2 | LMO2 | NT5C2 | SAMHD1 | TNFAIP3 |
| TP53 | TRAF3 | U2AF1 | UBA2 | WHSC1 | SETBP1 | TAL1 |
| XPO1 | ZEB2 | ZRSR2 | WT1 | TLX3 | XBP1 |  |
